# Preliminary Investigation of Potential Early Biomarkers for Gestational Diabetes Mellitus: Insights from *PTRPG* and *IGKV2D-28* Expression Analysis

**DOI:** 10.3390/ijms251910527

**Published:** 2024-09-30

**Authors:** Mariejim Diane Payot, Adrian Villavieja, Maria Ruth Pineda-Cortel

**Affiliations:** 1 The Graduate School, University of Santo Tomas, España Boulevard, Manila 1015, Philippines; mbpineda-cortel@ust.edu.ph; 2 Department of Medical Technology, University of Santo Tomas, España Boulevard, Manila 1015, Philippines; aevillavieja@ust.edu.ph; 3 Research Center for the Natural and Applied Sciences, University of Santo Tomas, España Boulevard, Manila 1015, Philippines

**Keywords:** biomarker, diagnosis, gene expression, gestational diabetes mellitus

## Abstract

Gestational diabetes mellitus (GDM) poses significant health risks to both mothers and infants, emphasizing the need for early detection strategies to mitigate its impact. However, the existing diagnostic methods, particularly the oral glucose tolerance test (OGTT) administered in the second or third trimester, show limitations in the detection of GDM during its early stages. This study aimed to explore the potential of the genes Protein Tyrosine Phosphatase Receptor-type Gamma (*PTPRG*) and Immunoglobulin Kappa Variable 2D-28 (*IGKV2D-28*) as early indicators for GDM among Filipino pregnant women. Utilizing reverse transcription–quantitative polymerase chain reaction (RT-qPCR), the gene expressions were analyzed in first-trimester blood samples obtained from 24 GDM and 36 non-GDM patients. The diagnostic performance of *PTPRG* and *IGKV2D-28* was analyzed and evaluated using receiver operating characteristic (ROC) curves. The findings revealed elevated expression levels of *PTPRG* and *IGKV2D-28* within the GDM cohort. Remarkably, *PTPRG* exhibited a sensitivity of 83%, while *IGKV2D-28* demonstrated a specificity of 94% at determined cut-off values. Combining both genes yielded an improved but limited diagnostic accuracy with an area under the curve (AUC) of 0.63. This preliminary investigation of *PTPRG* and *IGKV2D-28* sheds light on novel avenues for early GDM detection. While these findings are promising, further validation studies in larger cohorts are necessary to confirm these results and explore additional biomarkers to enhance diagnostic precision in GDM pregnancies and, ultimately, to improve maternal and fetal outcomes.

## 1. Introduction

Gestational diabetes mellitus (GDM) is characterized by the presence of any degree of glucose intolerance that is either initiated or initially recognized during pregnancy [1]. It emerges as a frequent complication linked to unfavorable consequences for both maternal and fetal well-being, encompassing an elevated susceptibility to macrosomia, obesity, cardiovascular diseases, and increased morbidity and mortality in the fetus [2]. GDM exhibits a significant correlation with glycemic control during pregnancy, which elevates the likelihood of developing type 2 diabetes in the postpartum period [3].

International Diabetes Federation (IDF), reported in 2021, that approximately 21.1 million live births (16.7%) were associated with hyperglycemia during pregnancy, in which 80.3% were attributed to GDM [4]. Substantial evidence indicates that Asians exhibit an elevated prevalence of GDM, with rates ranging from 8.8% to 20.0% in the Middle East, 9.6% to 18.3% in Southeast Asia, and 4.5% to 20.3% in the Western Pacific region. However, comparing the prevalence across Southeast Asian countries or regions is challenging due to the existence of different diagnostic guidelines [5,6]. In Metro Manila, Philippines, the International Association of the Diabetes and Pregnancy Study Groups (IADPSG) criteria were utilized, resulting in the prevalence rate increasing from 5.22% (as per the WHO criteria) to 16.32% (as per ADA criteria) and reaching 29.27% [7]. This shift underscores the impact of varying diagnostic criteria on the reported GDM prevalence within the studied population.

GDM is typically diagnosed in the second or third trimester, particularly within 24 to 28 weeks of gestation, with an oral glucose tolerance test (OGTT) [8]. This period potentially misses the early stages of metabolic dysfunctions that could impact maternal and fetal health. Enhancing early detection is crucial, and integrating maternal risk factors with preceding biomarkers for insulin resistance may offer a more effective approach [9]. Currently, there are no methods available that are widely implemented in routine laboratory practice for identifying women at risk of developing GDM during the first trimester. This gap in early detection underscores the urgent need for innovative approaches to identify at-risk individuals early, enabling timely intervention and potentially improving outcomes [10]. There are recommendations concerning whether early screening and diagnosis of GDM can be refined by incorporating biomarkers associated with its pathophysiology [9]. 

In a previous next-generation sequencing (NGS) study, consistently elevated expression levels of two specific genes were observed in pregnant women with GDM. *Protein Tyrosine Phosphatase Receptor-type Gamma* (*PTPRG*), identified as a tumor suppressor gene, has been demonstrated to play a role in metabolic disorders such as diabetes [11]. *Immunoglobulin Kappa Variable 2D-28* (*IGKV2D-28*) was consistently overexpressed in the GDM cohort, even among individuals with a normal BMI. These suggest the potential of these genes to function as early indicators in the development of GDM [12].

In this study, we examined the *PTPRG* and *IGKV2D-28* gene expression levels in first-trimester blood samples, aiming to ascertain their relevance in pregnant Filipino women diagnosed with GDM compared to non-GDM. Our investigation extends to the validation of prior NGS findings, assessing the potential utility of these genes as early indicators for GDM in susceptible individuals. Through this study, we seek to contribute to the early detection strategies for GDM, particularly in the context of the Filipino population, offering valuable insights that may inform more effective diagnostic approaches and intervention measures.

## 2. Results

### 2.1. Increased PTPRG and IGKV2D-28 Expression Levels in the GDM and Non-GDM Groups

**Figure 1:**
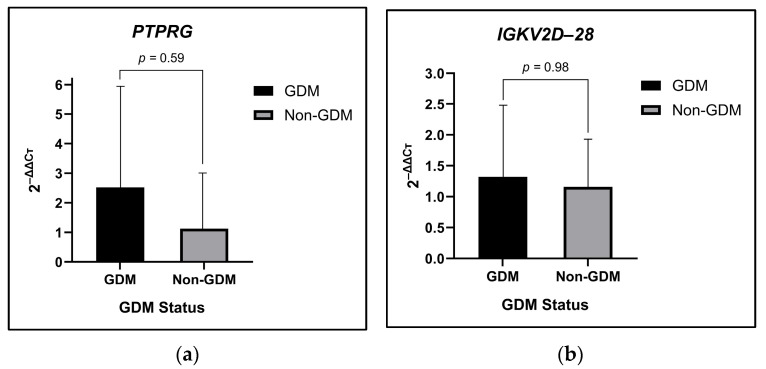
Levels of expression of the candidate genes (**a**) *PTPRG* and (**b**) *IGKV2D-28* in healthy pregnant women and those with GDM.

The gene expression levels of *PTPRG* and *IGKV2D-28* were assessed using primer probes through RT-qPCR, employing cDNA synthesized from first-trimester RNA samples. Analysis of the gene expression revealed that when compared to the samples in the non-GDM group, the samples in the GDM group showed higher mean ± SD values for both *PTPRG* (GDM 2.52 ± 3.27 vs. non-GDM 1.41 ± 1.46) and *IGKV2D-28* (GDM 1.32 ± 1.13 vs. non-GDM 1.16 ± 0.76), as illustrated in Figure 1. The observable differences in the gene expressions of both genes between both groups suggest the potential implications of measuring *PTPRG* and *IGKV2D-28* gene expressions in the pathophysiology of GDM; thus, further investigation into their roles as potential biomarkers for GDM diagnosis and management is warranted.

### 2.2. Correlations between Gene Expressions with Clinical Variables and GDM Status

The correlations between the expressions of the candidate genes and the clinical variables, such as the OGTT and HbA1c values, using the Spearman r test, are detailed in Table 1. Significant positive associations were observed between the *PTPRG* (r = 0.40; 95% CI 0.13 to 0.62; *p* = 0.01) and *IGKV2D-28* (r = 0.34; 95% CI 0.09 to 0.56; *p* = 0.01) expressions and the FBS levels. Consequently, GDM status was significantly positively correlated with the 2^−ΔΔCT^ values of *PTPRG* (r = 0.31; *p* = 0.03) and *IGKV2D-28* (r = 0.32; *p* = 0.01). These findings suggest a potential interaction between the expressions of the candidate genes and blood sugar levels, highlighting their possible role in GDM.

### 2.3. Diagnostic Potential of PTPRG and IGKV2D-28 in GDM Prediction

**Figure 2:**
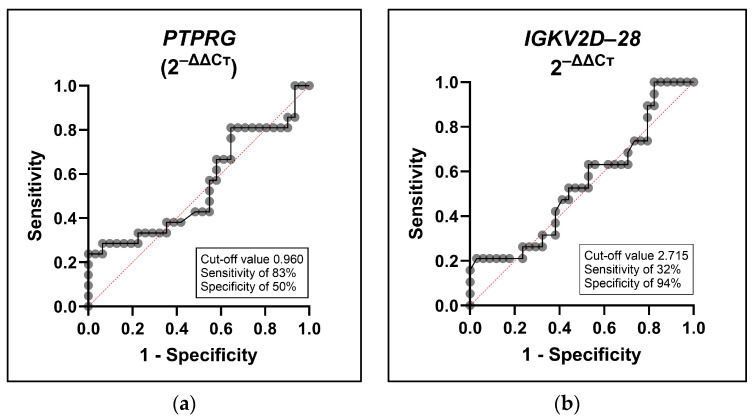
Receiver operating characteristic (ROC) curve analyses for differential gene expressions of (**a**) *PTPRG* and (**b**) *IGKV2D-28* to discriminate GDM patients from healthy pregnant women. The plotted points (indicated by black circles) represent various thresholds determined by the true positive rate (TPR) and the false positive rate (FPR). The red dotted lines divide the graph into two equal sections. The TPR, also referred to as sensitivity, is represented on the y-axis, while the FPR, defined as 1 minus specificity, is shown on the x-axis. Connecting these points creates a curve (represented by a solid black line) that illustrates the relationship between sensitivity and specificity. The AUC serves as a quantitative measure of the classifier’s ability to distinguish between positive and negative outcomes, thus providing a comprehensive assessment of its overall performance.

The effectiveness of employing the expression levels of the two candidate genes to classify GDM was assessed through ROC analysis. As demonstrated in Figure 2, both *PTPRG* (AUC 0.55; 95% CI 0.38 to 0.71; *p* = 0.58) and *IGKV2D-28* (AUC 0.54; 95% CI 0.38 to 0.70; *p* = 0.61) exhibited limited discriminatory accuracy. These findings suggest that the candidate genes alone may not serve as sufficient biomarkers for GDM diagnosis.

From the ROC curve analysis of each gene, the Youden J statistic was calculated for every plotted value using the formula: (Sensitivity + Specificity − 1). The plotted value exhibiting the highest Youden J statistic was identified as the optimal cut-off value for each gene, as also shown in Figure 2. Subsequently, every measured value was compared to its respective cut-off point to determine whether it was positive or negative. These diagnostic statuses were then cross-referenced with the participants’ GDM status determined by OGTT.

The values identified as positive for both the cut-off value and OGTT were categorized as true positives (TPs), while those negative for both were designated as true negatives (TNs). The instances where values were positive for the cut-off value but not for OGTT were considered false positives (FPs), and conversely, the values positive for OGTT but not for the cut-off point were denoted as false negatives (FNs). These tallies were then utilized to calculate the diagnostic sensitivity (TP/[TP + FN]) and diagnostic specificity (TN/[TN + FP]) for each gene.

**Figure 3:**
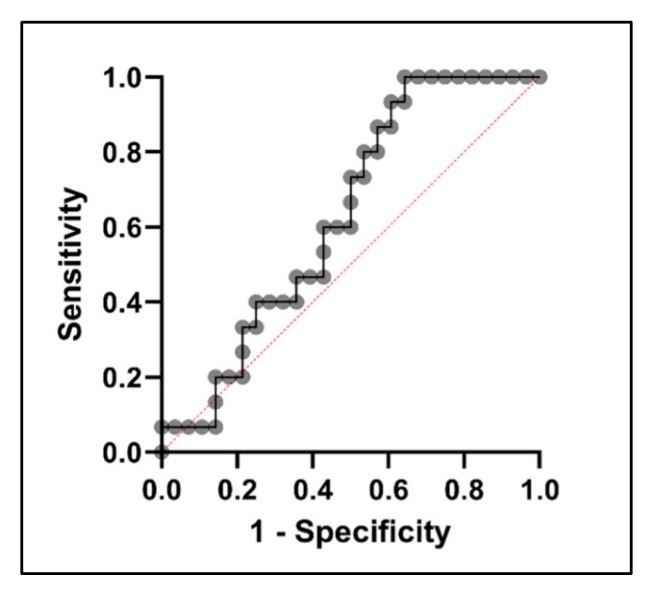
Receiver operating characteristic (ROC) curve analyses for combined measurements of *PTPRG* and *IGKV2D-28* to discriminate GDM patients from healthy pregnant women. The plotted points (indicated by black circles) represent various thresholds determined by the true positive rate (TPR) and the false positive rate (FPR). The red dotted lines divide the graph into two equal sections. The TPR, also referred to as sensitivity, is represented on the y-axis, while the FPR, defined as 1 minus specificity, is shown on the x-axis. Connecting these points creates a curve (represented by a solid black line) that illustrates the relationship between sensitivity and specificity. The AUC serves as a quantitative measure of the classifier’s ability to distinguish between positive and negative outcomes, thus providing a comprehensive assessment of its overall performance.

Utilizing these values, the diagnostic sensitivity and specificity of each gene were calculated. Alone, *PTPRG* exhibited notable sensitivity at 83%, whereas *IGKV2D-28* demonstrated a high specificity at 94%. Although each gene may not serve as an optimal standalone indicator, the potential of combining the measurements of both genes can be further investigated for improved GDM diagnosis. To explore this potential, multiple logistic regression was performed, combining the effects of both *PTPRG* and *IGKV2D-28*, with the odds ratio of 2.039 (*p* = 0.14). When combining the two genes, the diagnostic performance improves, as suggested by the AUC of 0.63, as seen in Figure 3.

## 3. Discussion

GDM presents many challenges that demand comprehensive exploration and understanding. The complexities associated with GDM range from variations in screening methodologies [9] and lack of consensus among healthcare professionals on the determination of the precise threshold for diagnosis [13] to the absence of universally accepted diagnostic criteria [14]. These challenges not only impact the accurate definition and prevalence estimation of GDM, particularly in Southeast Asian countries [15], but also extend to its subsequent management and the implications for maternal and fetal health, leading to higher rates of health problems and even death [10]. The lack of universally accepted gold standards for GDM screening has resulted in diverse approaches to identifying GDM, affecting the precision of prevalence estimates [16].

This study showed that *PTPRG* and *IGKV2D-28* could potentially identify pregnancies at risk for GDM as early as the first trimester. Detection during the first trimester period provides an opportunity to explore possible preventive measures and treatment strategies for pregnant women. According to a 2021 study, following differential expression-based hub gene identification, the genes *PTPRG* and *IGKV2D-28* exhibited the highest expression levels in the GDM group compared to non-GDM groups [12]. This pattern was confirmed in our current study using first-trimester blood samples collected from Filipino pregnant women.

*PTPRG* is a member of the protein tyrosine phosphatase (PTP) family. It is known to regulate signaling molecules that are important in cell growth, differentiation, the mitotic cycle, and oncogenic transformation. It is located on chromosome 3p14.2/21, with its messenger RNA encoding a transmembrane protein comprising three distinct domains within the extracellular amino terminus region and spanning 5787 base pairs. PTPs are a large family of enzymes that are essential regulators in signal transduction pathways [17]. They play a significant role in maintaining diverse cellular functions. They act by catalyzing the removal of phosphate groups from protein residues. Dysregulation of PTP activity is implicated in the pathogenesis of various disorders, such as neurological conditions and autoimmune disorders [18]. Moreover, when PTP activity is defective or improperly regulated, it results in aberrant tyrosine phosphorylation, thereby contributing to the development of diseases such as cancer and diabetes [17,19].

One important role of PTP is insulin signaling, a critical process in cellular metabolism and glucose regulation that modulates insulin receptor activity by dephosphorylating and inactivating it [20]. Our results show an increase in *PTPRG* in patients with GDM, which could probably be linked to the inactivation of insulin receptors, causing increased blood glucose levels. Maternal insulin resistance is one of the central features of pregnancies complicated by GDM. It amplifies the demand for insulin secretion, leading to hyperinsulinemia and subsequent metabolic disturbances [21]. Insulin, an anabolic hormone secreted by pancreatic β-cells, facilitates glucose uptake into peripheral tissues to regulate glucose homeostasis [22]. However, insulin resistance disrupts this finely tuned process and manifests when cells exhibit diminished responsiveness to insulin’s regulatory effects. At the molecular level, this resistance typically arises from impaired insulin signaling pathways [23]. *PTPRG* functions by dephosphorylating phosphotyrosine residues on specific intracellular targets [24]. For instance, the hepatic expression of *PTPRG* is known to be upregulated in response to inflammation in obese and type 2 diabetes mellitus (T2DM) mouse models, aligning with findings in humans where elevated *PTPRG* expression positively correlates with markers of inflammation and insulin resistance [25]. In a study involving a Korean population, an integrated predictive model for T2DM was developed, merging clinical and genetic factors based on single-nucleotide polymorphism (SNP) point scores. The analysis revealed five key SNPs, with rs9311835 located on the *PTPRG* intron emerging as a potential predictive marker [26]. Another member of the PTP family is PTP1B, which has been studied as a target for diabetic drugs. Their results showed that metformin could inhibit PTP1B and improve glucose regulation in mouse models [27].

The other target gene in this study is *IGKV2D-28*. It is contextualized within the broader framework of immunoglobulin synthesis and immune system function. Immunoglobulins, consisting of two identical heavy chains and two identical light chains connected by disulfide bonds, are crucial for immune function. These structures, which are pivotal for immune responses, are generated by B cells, which also produce kappa (κ) and lambda (λ) free light chains (FLCs), contributing to immunoglobulin synthesis and immune modulation [28]. The gene *IGKV2D-28*, located on chromosome 2 within the immunoglobulin kappa (IGK) locus, contributes to the intricate network of immune system regulation [29]. 

The relevance of *IGKV2D-28* is explored within the context of GDM, which mirrors the complex pathology of T2DM, which is marked by insulin resistance and inadequate insulin secretion. While the specific pathophysiological mechanisms underlying diabetic pregnancies remain elusive, emerging evidence highlights the immune system dysregulation and inflammatory processes as critical contributors to the pathophysiology of GDM [30]. Our results show an increased gene expression of *IGKV2D-28* in the GDM group compared to the non-GDM group, which could be associated with immune dysregulation and inflammation in GDM. Maternal hyperglycemia triggers inflammation, with both placental and visceral adipose tissue contributing to the inflammatory response, which involves altered infiltration, differentiation, and activation of maternal innate and adaptive immune cells. This dysregulation of maternal immune function exacerbates the condition, further reducing maternal insulin sensitivity [31]. 

In T2DM, immunoglobulin FLCs were found to be promising inflammatory biomarkers. Notably, the FLC kappa/lambda ratio demonstrated remarkable diagnostic performance, with a sensitivity of 96% and a specificity of 100% compared to conventional markers such as HbA1c [32]. Investigations into diabetic nephropathy have identified serum FLCs as potential early markers, further emphasizing their utility in disease diagnosis and management [33]. Through these findings, the multifaceted roles of immunoglobulin-related components, including *IGKV2D-28* and FLCs, in immune system modulation and disease pathogenesis were elucidated.

Currently, at the time of the present study, there is no literature investigating the association between *IGKV2D-28* and GDM. Despite the growing body of research on genetic biomarkers in GDM, the specific role of *IGKV2D-28* remains unexplored, highlighting the need for further investigation into its potential implications in GDM pathogenesis and diagnosis.

Several recommendations from the findings of this study can be proposed to enhance the diagnosis and management of GDM. To our knowledge, our investigation represents the first attempt to validate the initial results of the previous NGS study [12], which identified potential candidate genes for early GDM detection. While NGS was previously conducted to identify these candidate genes, in the present study, we focused on validating and analyzing their expression using the same set of samples. Given the preliminary nature of these findings, we recommend that further studies confirm these results in a new cohort of pregnant women to establish broader clinical relevance. Additionally, research involving a larger cohort is essential to more comprehensively evaluate the diagnostic performance and potential of these genetic biomarkers.

It is also essential to investigate additional biomarkers that can complement the diagnostic accuracy of *PTPRG* and *IGKV2D-28*, potentially leading to the development of a more robust diagnostic panel for GDM. More validation studies should be conducted to assess the effectiveness of combining multiple biomarkers in improving diagnostic sensitivity and specificity. Considering the limitations associated with OGTT, delving into alternative diagnostic approaches, such as utilizing biomarkers from first-trimester blood samples, could offer valuable insights.

Another limitation of this study is that, although gene expression analysis provided preliminary understanding of the potential roles of *PTRPG* and *IGKV2D-28* in GDM pathogenesis, the measured mRNA levels may not directly correlate with protein synthesis. Nonetheless, gene expression studies still serve as a critical starting point for identifying relevant pathways. The findings of this study highlight promising areas for further exploration and underscore the need for the incorporation of complementary approaches to confirm the relevance of these genes in GDM.

Our initial findings are focused on pregnant women as a general population, without distinguishing between specific high-risk groups. As we have not yet stratified or characterized the study population into distinct risk categories, our main objective was to explore potential biomarkers for early GDM detection across a broader spectrum of pregnant women. We acknowledge that future studies should assess whether these biomarkers are more relevant to high-risk groups or could be applied universally. Moving forward, further research will be needed to stratify the population and evaluate whether these markers demonstrate particular significance among high-risk subgroups, such as women with a family history of diabetes, obesity, or prior GDM.

The traditional OGTT, though effective, primarily identifies GDM once hyperglycemia has developed, which may limit the window for early intervention. In contrast, the biomarkers identified in our study offer the potential for earlier detection. This could allow more timely and personalized interventions, ultimately improving maternal and fetal outcomes. Although cost considerations remain, the findings from this study may contribute to the development of more refined and cost-effective diagnostic tools in the future, with the potential for significant long-term clinical and economic benefits.

## 4. Materials and Methods

### 4.1. First-Trimester RNA Samples

A total of 60 first-trimester RNA eluates from 24 patients diagnosed with GDM (GDM group) and 36 patients without GDM (non-GDM group) were processed. The sample size was calculated using G*Power version 3.1.9.7, employing a two-tailed test with an alpha level of 0.05, a statistical power of 0.80, and an effect size of 0.8. As presented in Table 2, their GDM status was pre-identified using OGTT values that were part of a previous GDM study, with ethical clearance from the Ethics Review Committee of the University of Santo Tomas Graduate School (Protocol Number: E-2016-02-R3).

Prior to the analysis of the gene expression, total RNA from blood collected in Tempus™ Blood RNA Tubes (Applied Biosystems, Foster City, CA, USA) was extracted using the Tempus™ Spin RNA Isolation Kit (Applied Biosystems, Foster City, CA, USA), following the manufacturer’s instructions. The quality and quantity of the RNA were assessed. Absorbance at 260 and 280 nm was read using Nanodrop™ FLUOstar^®^ Omega Microplate Reader (BMG LABTECH, Ortenburg, Germany). RNA concentration was measured using a Qubit RNA Broad-Range Assay kit (Applied Biosystems, Foster City, CA, USA) in a Qubit 4.0 fluorometer.

### 4.2. Reverse Transcription and cDNA Synthesis

The first-strand cDNA was synthesized from cell RNA using the SuperScript^®^ III First-Strand Synthesis System (Catalog No. 18080051, Invitrogen, Carlsbad, CA, USA), following the manufacturer’s instructions with slight modifications. Briefly, 5 µL of isolated RNA (mg) from the cells was mixed with 1 µL of random hexamer primer, 1 µL of 10 mM dNTP mix, and 3 µL of DEPC-treated water. This was followed by incubation at 65 °C for 5 min and chilling on ice for at least 1 min. Afterwards, a 10 µL of cDNA synthesis mixture of 2 µL of 10X RT buffer, 4 µL of 25 mM MgCl_2_, 2 µL of 0.1 M DTT, 1 µL RNaseOUT (40 U/uL), and 1 µL SuperScript III RT was added to each sample. Immediately, the samples were incubated at 25 °C for 10 min, followed by 50 °C for 50 min, and the reaction was terminated by heating at 85 °C for 5 min. Then, 1 µL of RNase H was added to each tube to remove the RNA and was incubated at 37 °C for 20 min. Reverse transcription was performed in the final volume of 21 µL.

### 4.3. Real-Time Quantitative RT-PCR

Commercially available predesigned TaqMan^®^ Gene Expression Assays (*PTPRG*, Hs00892788_m1; GAPDH, Hs03929097, Applied Biosystems, Foster City, CA, USA) with probes labeled with 6-FAM™ dye-labeled TaqMan^®^ MGB and TaqMan^®^ Fast Advanced Master Mix (Catalog No. 444457, Applied Biosystems, Foster City, CA, USA) were utilized in real-time PCR for relative quantification of *PTRPG* and *IGKV2D-28* genes. The primer-probe for *IGKV2D-28* (Forward: 5′-AGGTCTAGTCAGAGCCTCCT-3′; Reverse: 5′- GAGGCCCGATTAGAACCCAA-3′; Oligonucleotide: AGCCAGGGCAGTCTCCACAGCTCCTGA) was designed using the online NCBI Primer-Blast program (http://www.ncbi.nlm.nih.gov/tools/primer-blast/), accessed on 1 August 2023, and was produced by the Custom TaqMan™ Gene Expression Assay (Assay ID: APKCDYE; Catalog No. 4331348, Applied Biosystems).

Components were mixed according to the manufacturer’s protocol and amplified in 20 µL total volume/well (96-well plates) using a Rotor-Gene Q^®^ real-time PCR cycler (Qiagen, Hilden, Germany). Each reaction mixture contained 10 µL master mix, 1.0 µL primer-probe assay, 2 µL cDNA template, and 7 µL nuclease-free water. The quantitative RT-qPCR conditions were UNG incubation at 50 °C for 2 min, followed by polymerase activation at 95 °C for 20 s, 40 cycles of denaturation at 95 °C for 1 s, and annealing and extension at 60 °C for 20 s.

### 4.4. Computation of Gene Expression

The cycle threshold (Ct) values were automatically generated by the Rotor-Gene Q Series Software (version 2.3.1), and relative gene expression was calculated and normalized by the standard 2^−ΔΔCT^ method, using the gene expression of *GAPDH* as a reference.

### 4.5. Statistical Analysis

All statistical analyses were performed using GraphPad Prism version 10.1.2 (GraphPad Software Inc., San Diego, CA, USA). Differences in gene expression values (2^−ΔΔCT^) between the two study groups were compared using the Mann–Whitney U test. Spearman rank correlation analysis was initially conducted to evaluate the relationship between the gene expression and OGTT parameters (glucose, 1st hour, and 2nd hour). Logistic regression analysis was then employed to assess the association between the gene expression and GDM status (GDM vs. non-GDM). To evaluate the diagnostic potential of both *PTPRG* and *IGKV2D-28* gene expression, receiver operating characteristic (ROC) analysis was performed to obtain the AUC, followed by the identification of the cut-off values using Youden’s index. The diagnostic sensitivity and specificity of the gene expression values were subsequently computed based on the determined cut-off values. Multiple logistic regression analysis was conducted to evaluate the diagnostic potential of measuring *PTPRG* and *IGKV2D-28*. The statistical significance was set at *p* < 0.05.

## 5. Conclusions

In this study, we conducted a preliminary investigation aimed at identifying potential diagnostic biomarkers for the early detection of GDM. Our findings revealed that *PTPRG* and *IGKV2D-28* exhibited elevated expression levels in the first-trimester blood samples from pregnant women with GDM. The diagnostic sensitivity and specificity of *PTPRG* (83%) and *IGKV2D-28* (94%) were notable, and their combined measurement showed an improved diagnostic performance with an AUC of 0.63. These results underscore the complex nature of GDM diagnosis and highlight the need for further research to identify additional biomarkers for more accurate detection and management of GDM. This holds the potential to prevent maternal and fetal complications and emphasizes the importance of early GDM detection. Moreover, the challenges associated with the OGTT, such as the lack of universal diagnostic criteria and the inconvenience of the procedure, further emphasize the importance of identifying alternative diagnostic approaches. Future studies should focus on elucidating the molecular mechanisms underlying GDM pathogenesis and exploring novel strategies for early detection and intervention, ultimately improving maternal and fetal outcomes in pregnancies complicated by GDM.

## Figures and Tables

**Table 1:** Correlation of *PTPRG* and *IGKV2D-28* gene expressions with clinical variables and GDM status.

Candidate Genes	Glucose		1st Hour OGTT		2nd Hour OGTT		HbA1c		GDM Status	
	r	*p* Value	r	*p* Value	r	*p* Value	r	*p* Value	r	*p* Value
*PTPRG* (2^−ΔΔCT^)	0.40 ^b^	0.01 ^a^	−0.02 ^b^	0.88	−0.21 ^b^	0.17	0.18 ^b^	0.23	0.31 ^b^	0.03 ^a^
*IGKV2D-28* (2^−ΔΔCT^)	0.34 ^b^	0.01 ^a^	−0.08 ^b^	0.57	−0.14 ^b^	0.34	0.21 ^b^	0.15	0.32 ^b^	0.01 ^a^

OGTT: oral glucose tolerance test; HbA1c: glycated hemoglobin; GDM: gestational diabetes mellitus; r: Spearman’s correlation coefficient; 2^−ΔΔCT^: relative fold gene expression. ^a^ Significant at *p* < 0.05 compared with the candidate genes. ^b^ The r values > 0 suggest a direct (positive) relationship, while the r values < 0 suggest an inverse (negative) relationship.

**Table 2:** OGTT results of pregnant women with and without gestational diabetes mellitus.

Biochemical Characteristics	Non-GDM (*n* = 36)	GDM (*n* = 24)
Glucose (mmol/L)	4.49 ± 0.39	5.51 ± 0.78
First hour OGTT (mmol/L)	6.29 ± 0.83	8.55 ± 1.75
Second hour OGTT (mmol/L)	6.28 ± 0.98	7.69 ± 1.88

GDM: gestational diabetes mellitus; OGTT: oral glucose tolerance test.

## Data Availability

The raw data supporting the conclusions of this article will be made available by the authors on request because data of this study are part of an on-going study. Request to such can be directed to the corresponding author.
